# Supplementary material for: Defining the impact of dietary macronutrient balance on PCOS traits
Source: Nat Commun. 2020 Oct 16;11:5262. doi: 10.1038/s41467-020-19003-5 (PMC7568581; doi:10.1038/s41467-020-19003-5)
Supplement: Supplementary file 1 — Supplementary Information [file 41467_2020_19003_MOESM1_ESM.pdf]

Supplementary information

Defining the impact of dietary macronutrient balance on PCOS traits

Rodriguez Paris et al.

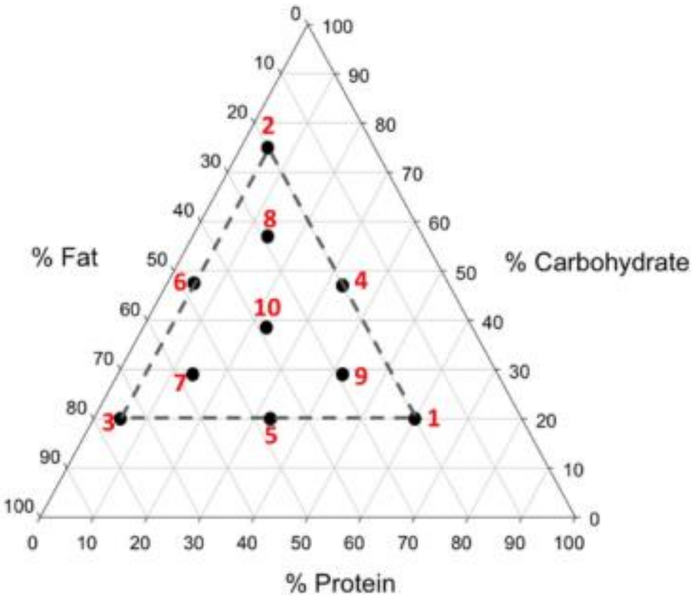

Supplementary Fig. 1
Reasoning for selection of macronutrient content of experimental diets. The red numbers indicate one of the ten diets outlined in Table S1. The macronutrient composition of the experimental diets are confined to the black doted triangle with energy composition ranging from: 5-60% protein, 20-75% carbohydrate and 20-75% fat. The diet combinations within the triangle have been selected for optimal power in fitting response surface models.

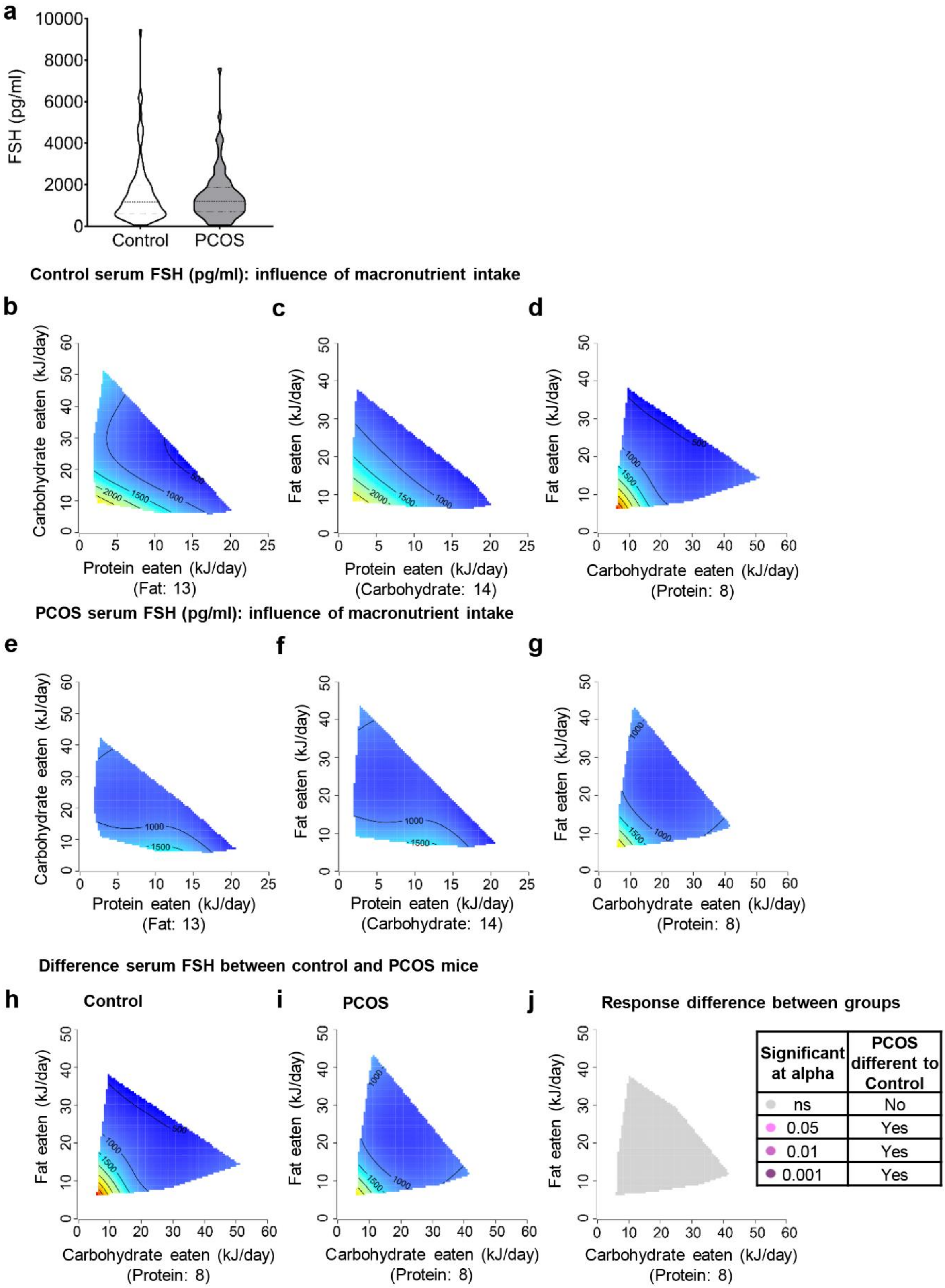

Supplementary Fig. 2

FSH levels in PCOS mice are comparable to control mice. **a**, Serum FSH levels, showing that irrespective of diet androgen excess did not alter serum FSH levels. Data presented as violin plots, doted lines indicate the median and dashed lines the 25% and 75% percentiles. **b-g**, 3D GAM response surfaces displaying the relationship between macronutrient intake (kJ/day) and serum FSH levels in control (**b-d**) and PCOS mice (**e-g**). Red areas indicate the

greatest value for each response, which then decreases to the lowest value as the colour shifts to blue. Response surfaces display an overall blue colour in both control and PCOS mice indicating similar ranges of serum FSH levels. However, in both control and PCOS mice statistical analysis revealed that the interaction of C and F intake significantly influenced FSH levels, with low C and F intake leading to higher FSH levels. **h-j**, Response surfaces showing the effects of C and F intakes on serum FSH levels in control mice (**h**), PCOS mice (**i**) mice and response difference (**j**), indicating that serum FSH levels in PCOS mice are comparable to control mice. **a-j,** n = 88 control and 92 PCOS mice.

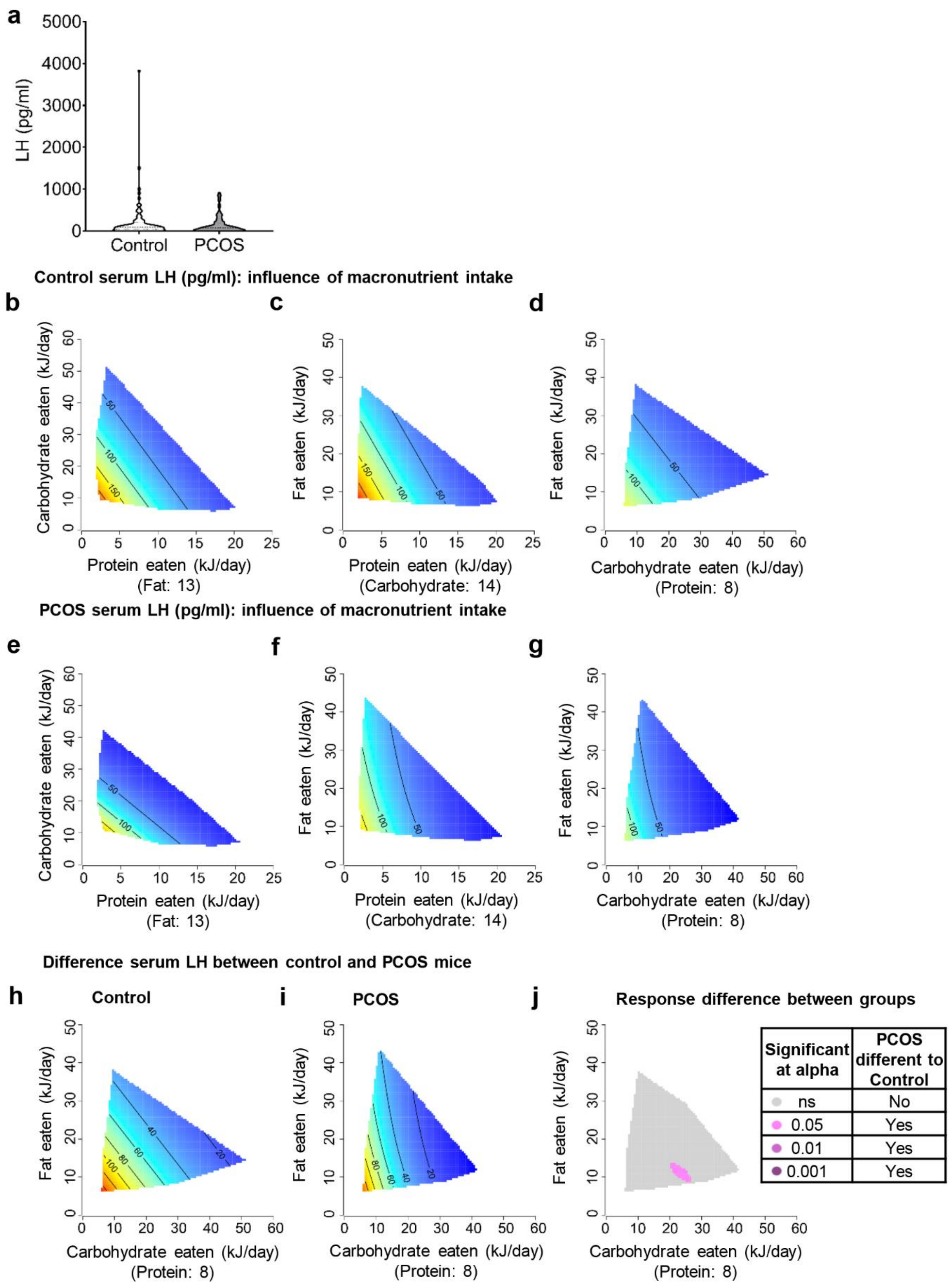

Supplementary Fig. 3

LH levels in PCOS mice are comparable to control mice. **a**, Serum LH levels, showing that irrespective of diet androgen excess did not alter serum LH levels. Data presented as violin plots, doted lines indicate the median and dashed lines the 25% and 75% percentiles. **b-g**, 3D GAM response surfaces displaying the relationship between macronutrient intake (kJ/day) and

serum LH in control (**b-d**) and PCOS mice (**e-g**). Red areas indicate the greatest value for each response, which then decreases to the lowest value as the colour shifts to blue. Response surfaces show that P significantly influenced LH levels in control mice (**b and c**), while macronutrient intakes had no effect in PCOS mice (**e-g**). **h-j**, Response surfaces showing the effects of C and F intakes on serum LH levels in control mice (**h**), PCOS mice (**i**) and response difference (**j**), demonstrating that serum LH levels in PCOS mice are comparable to control mice. **a-j,** n = 88 control and 92 PCOS mice.

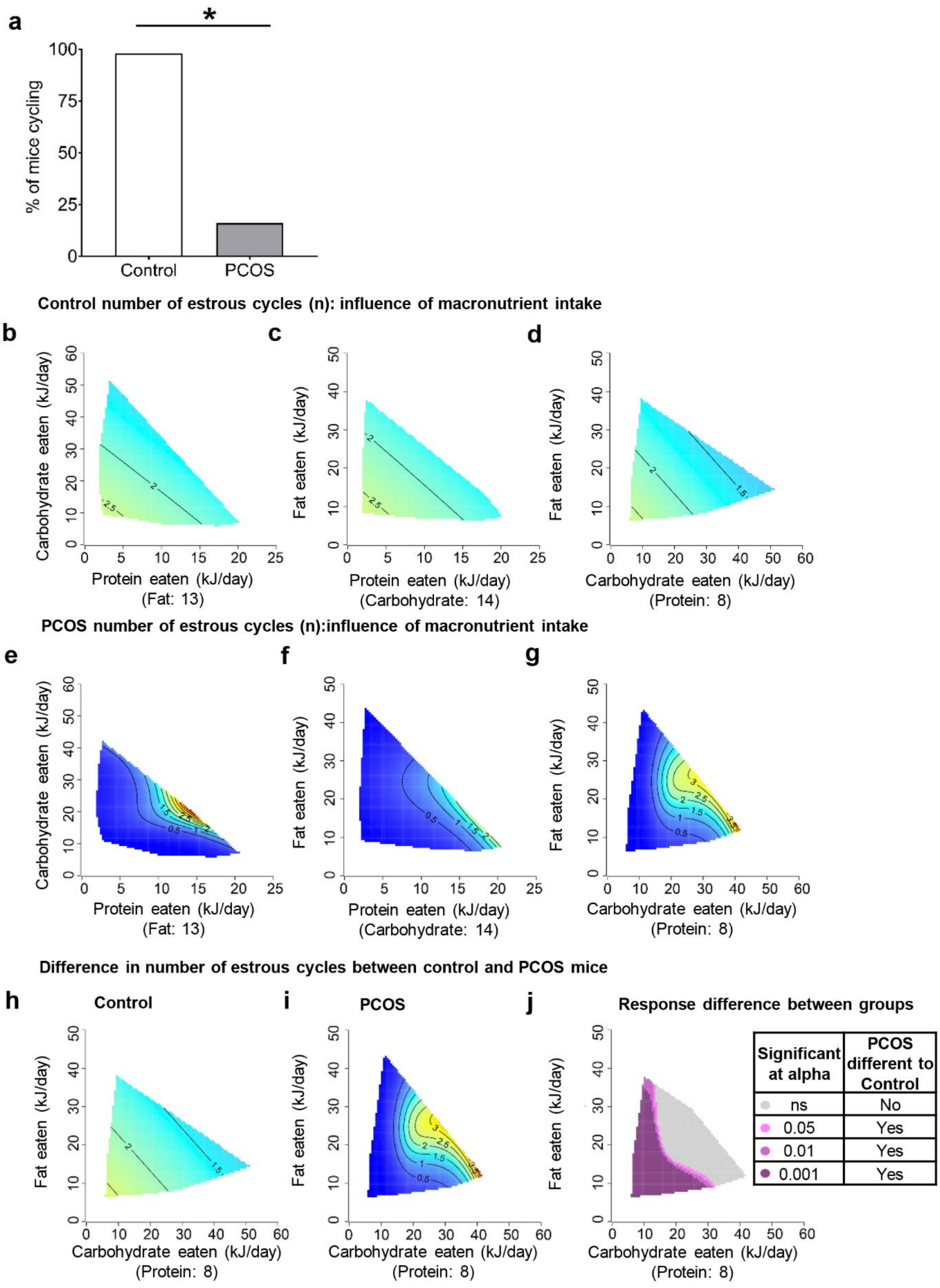

Supplementary Fig. 4

An optimal dietary macronutrient balance rescues estrous cyclicity in a PCOS mouse model. a, % of control and PCOS mice cycling, showing that a subset (16%) of PCOS mice exhibited restored estrous cycles (P<0.0001). Significance determined by Fisher's exact test,

asterisk (*) indicates P-value <0.05, n=94 control and 94 PCOS mice. **b-g,** 3D GAM Response surfaces displaying the relationship between macronutrient intake (kJ/day) and number of estrous cycles in control **(b-d)** and PCOS mice **(e-g)**. Red areas indicate the greatest value for each response, which then decreases to the lowest value as the colour shifts to blue. Analysis of response surfaces show that no specific macronutrient had a significant influence over the number of estrous cycles in control mice **(b-d)**, while the combination of P and C intake were the main drivers in PCOS mice **(e)**. **h-j,** 3D GAM response surfaces showing the effects of C and F intakes on number of estrous cycles in control mice **(h)**, PCOS mice **(i)** and response difference **(j)**, demonstrating a comparable estrous cycle response between control and PCOS when intakes were >20 kJ/day for C and > 15 kJ/day for F. **b-j,** n = 93 control and 94 PCOS mice.

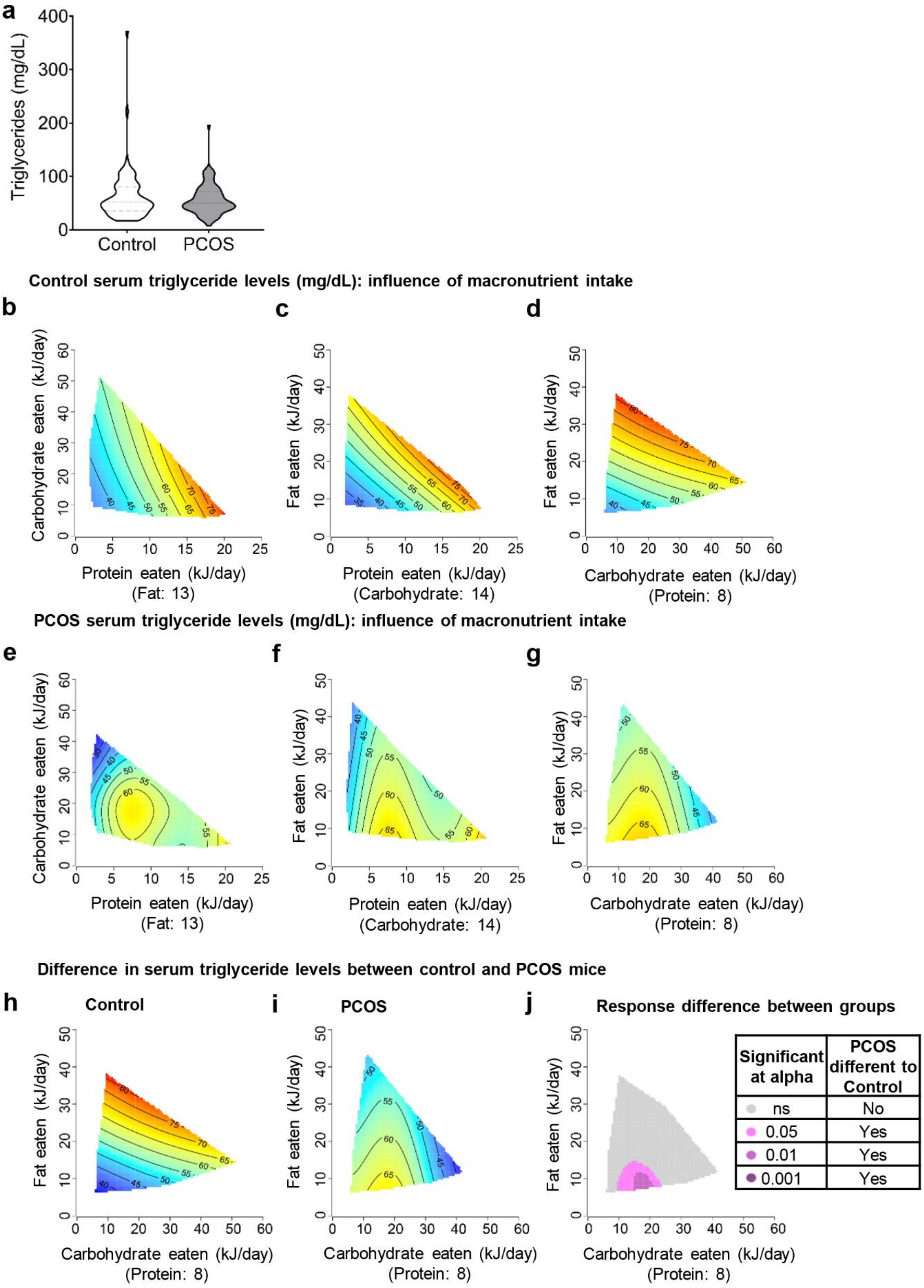

Supplementary Fig. 5

Triglyceride levels in PCOS mice are comparable to control mice. a, Serum triglyceride levels, showing that regardless of diet androgen excess did not induce the PCOS feature of elevated serum triglyceride levels. Data presented as violin plots, doted lines indicate the median and dashed lines the 25% and 75% percentiles. **b-g,** 3D GAM Response surfaces

displaying the relationship between macronutrient intake (kJ/day) and serum triglyceride levels in control **(b-d))** and PCOS mice **(e-g)**. Red areas indicate the greatest value for each response, which then decreases to the lowest value as the colour shifts to blue. Analysis of response surfaces show that P intake influenced triglyceride levels in control mice **(b and c)**, while the combination of P and C intake was the main driver in PCOS mice **(e)**, but overall levels are within a similar range for both groups (40-65 mg/dL). **h-j,** 3D GAM response surfaces showing the effects of C and F intakes on serum triglyceride levels in control mice **(h)**, PCOS mice **(i)** mice and response difference **(j)**, confirming that overall triglyceride levels in PCOS and control mice were comparable and displayed a similar response to macronutrient intake. **a-j,** n = 89 control and 94 PCOS mice.

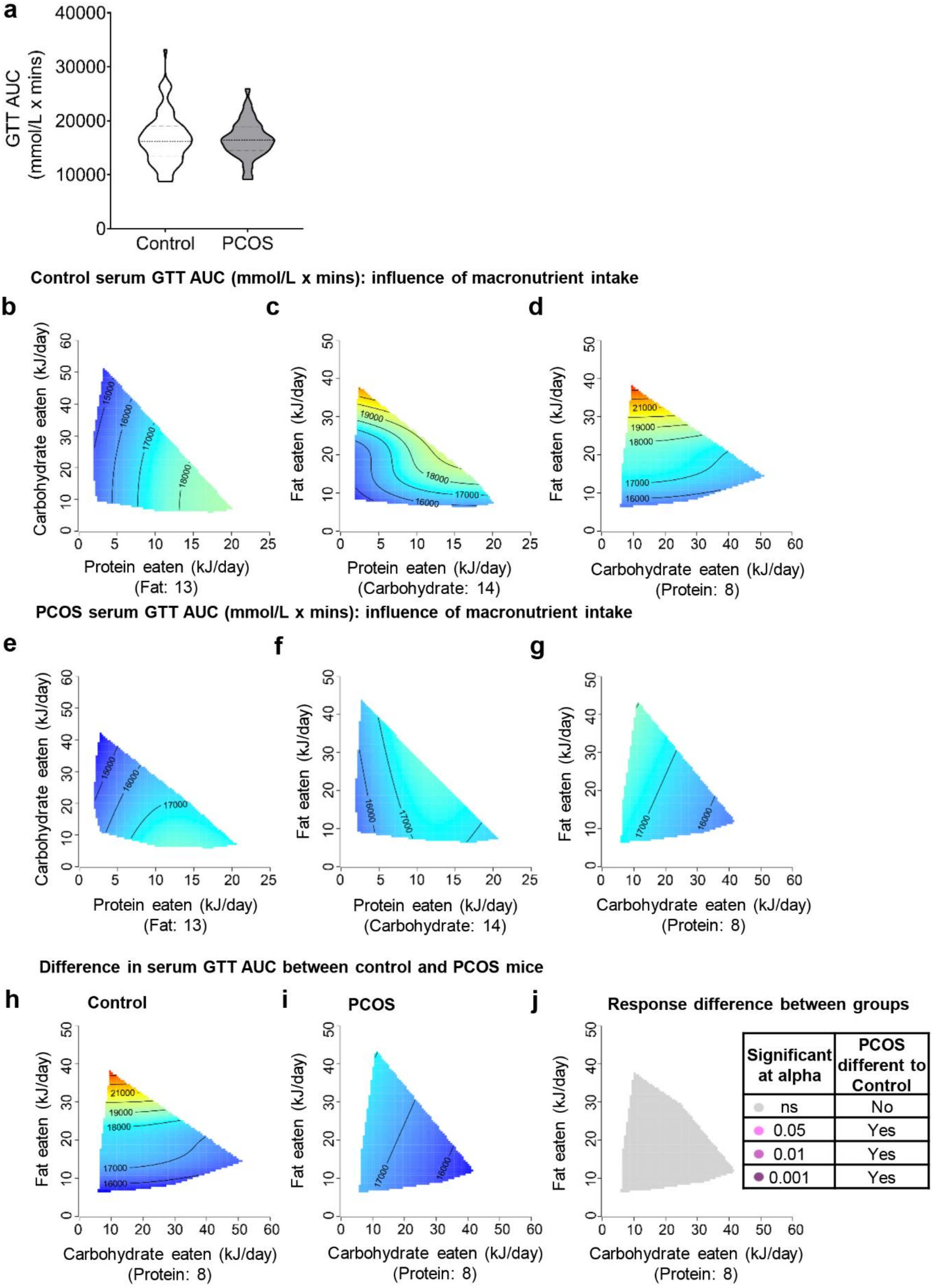

Supplementary Fig. 6

GTT AUC levels in PCOS mice are comparable to control mice. a, Average area under the curve (AUC) analysis of the glucose tolerance test (ipGTT), showing no significant increase in

GTT AUC levels in PCOS mice compared to control mice. Data presented as violin plots, doted lines indicate the median and dashed lines the 25% and 75% percentiles. **b-g,** 3D GAM response surfaces displaying the relationship between macronutrient intake (kJ/day) and GTT AUC in control **(b-d)** and PCOS mice **(e-g)**. Red areas indicate the greatest value for each response, which then decreases to the lowest value as the colour shifts to blue. Response surfaces show that F intake influenced GTT AUC in control mice **(c and d)**, while macronutrient intakes had no effect in PCOS mice **(e-g)**, but across the majority of dietary intakes both groups displayed similar GTT AUC. **h-j,** 3D GAM response surfaces showing the effects of C and F intakes on GTT AUC in control mice **(h)**, PCOS mice **(i)** and response difference **(j)**, demonstrating that across all diets GTT AUC response in PCOS mice was comparable to control mice. **a-j,** n = 93 control and 94 PCOS mice.

Supplementary Table 1

Macronutrient composition of the 10 diets. The % protein (P), carbohydrate (C) and fat (F) were calculated as a % of the total energy for each diet. The sources for protein were casein and methionine, for carbohydrate sucrose, wheat starch and dextrinized cornstarch and for fat soya bean oil. Other ingredients, such as cellulose, were kept to similar proportions. All diets were supplemented with a vitamin mix (vitamin A, D3, E, K, C, B1, B2, Niacin, B6, pantothenic acid, biotin, folic acid, inositol, B12 and choline) and mineral mix (Ca, P, Mg, Na, C, K, S, Fe, Cu, I, Mn, Co, Zn, Mo, Se, Cd, Cr, Li, B, Ni and V) to levels found in AIN-93G. **(a)** Diet 3 was discontinued after 6 control and 6 PCOS mice experienced a weight loss of ≥ 20% or failed to thrive.

**Diet**		**1**	**2**	**3**[a]	**4**	**5**	**6**	**7**	**8**	**9**	**10**
%P		60	5	5	33	33	5	14	14	42	23
%C		20	75	20	47	20	48	29	57	29	38
%F		20	20	75	20	47	48	57	29	29	38
**Energy**	**P**	8.88	0.74	0.75	4.84	4.93	0.74	2.09	2.09	6.27	3.43
(kJ/g)	**C**	3.03	11.62	3.06	7.31	3.06	7.46	4.47	8.87	4.47	5.89
	**F**	3.22	3.23	12.19	3.19	7.80	7.80	9.26	4.68	4.72	6.19

Supplementary Table 2. Significance of non-parametric smooth terms from GAMs associated with PCOS features. n = sample size (number of mice).

Response measured	edf	Ref.df	Chi.sq	p-value
**Corpora lutea/ovary and macronutrient intake**				
Control (n = 36)				
s(intake.P)	1.000	1.000	0.289	0.591
s(intake.C)	1.000	1.000	2.679	0.102
s(intake.F)	2.076	2.425	5.970	0.047
s(intake.P,intake.C)	2.603	3.000	11.434	0.004
s(intake.P,intake.F)	0.000	3.000	0.000	0.200
s(intake.C,intake.F)	0.001	3.000	0.001	0.033
PCOS (n = 35)				
s(intake.P)	1.000	1.000	0.017	0.896
s(intake.C)	1.000	1.000	0.082	0.775
s(intake.F)	1.000	1.000	0.092	0.761
s(intake.P,intake.C)	0.000	3.000	0.000	0.655
s(intake.P,intake.F)	0.000	3.000	0.000	0.691
s(intake.C,intake.F)	2.178	3.000	4.420	0.113
**Body weight (g) and macronutrient intake**				
Control (n = 93)				
s(intake.P)	2.433	2.689	14.993	0.000
s(intake.C)	1.000	1.000	4.880	0.030
s(intake.F)	1.000	1.000	7.160	0.009
s(intake.P,intake.C)	2.448	3.000	3.729	0.000
s(intake.P,intake.F)	1.923	3.000	3.982	0.000
s(intake.C,intake.F)	1.524	3.000	2.469	0.002
PCOS (n = 94)				
s(intake.P)	2.794	2.926	10.935	0.000
s(intake.C)	1.000	1.000	6.293	0.014
s(intake.F)	2.430	2.644	4.371	0.009
s(intake.P,intake.C)	1.901	3.000	4.213	0.001
s(intake.P,intake.F)	0.278	3.000	0.107	0.036
s(intake.C,intake.F)	0.000	3.000	0.000	0.639
**Serum adiponectin (ng/ml) and macronutrient intake**				
Control (n = 78)				
s(intake.P)	2.218	2.591	3.369	0.032
s(intake.C)	1.000	1.000	10.026	0.002
s(intake.F)	1.000	1.000	3.611	0.061
s(intake.P,intake.C)	0.007	3.000	0.002	0.325
s(intake.P,intake.F)	0.000	3.000	0.000	0.983
s(intake.C,intake.F)	0.000	3.000	0.000	0.448
PCOS (n = 87)				
s(intake.P)	2.064	2.449	2.148	0.105
s(intake.C)	1.015	1.031	0.676	0.407
s(intake.F)	1.000	1.000	0.058	0.811
s(intake.P,intake.C)	0.001	3.000	0.000	0.360
s(intake.P,intake.F)	0.000	3.000	0.000	0.791
s(intake.C,intake.F)	0.001	3.000	0.000	0.420
**Serum cholesterol (mg/dL) and macronutrient intake**				
Control (n = 91)				
s(intake.P)	1.280	1.495	6.545	0.005
s(intake.C)	1.000	1.000	1.159	0.285
s(intake.F)	1.302	1.527	3.084	0.118
s(intake.P,intake.C)	0.000	3.000	0.000	0.529
s(intake.P,intake.F)	0.000	3.000	0.000	0.436
s(intake.C,intake.F)	0.000	3.000	0.000	0.671
PCOS (n = 89)				
s(intake.P)	1.000	1.000	0.500	0.481
s(intake.C)	1.000	1.000	2.411	0.124
s(intake.F)	1.000	1.000	29.795	0.000
s(intake.P,intake.C)	1.284	3.000	0.824	0.137
s(intake.P,intake.F)	0.000	3.000	0.000	0.514

s(intake.C,intake.F)	0.001	3.000	0.000	0.301
**Serum fasting glucose (mmol/L) and macronutrient intake**				
Control (n = 93)				
s(intake.P)	1.000	1.000	0.511	0.476
s(intake.C)	1.000	1.000	0.161	0.689
s(intake.F)	1.000	1.000	4.650	0.034
s(intake.P,intake.C)	0.000	3.000	0.000	0.474
s(intake.P,intake.F)	1.376	3.000	0.888	0.134
s(intake.C,intake.F)	0.000	3.000	0.000	0.817
PCOS (n = 94)				
s(intake.P)	1.000	1.000	5.717	0.019
s(intake.C)	1.000	1.000	2.239	0.138
s(intake.F)	2.616	2.894	7.001	0.000
s(intake.P,intake.C)	0.000	3.000	0.000	0.724
s(intake.P,intake.F)	0.000	3.000	0.000	0.443
s(intake.C,intake.F)	0.000	3.000	0.000	0.568
**Serum FSH (pg/ml) and macronutrient intake**				
Control (n = 88)				
s(intake.P)	1.000	1.000	5.640	0.020
s(intake.C)	1.000	1.000	2.424	0.123
s(intake.F)	1.000	1.000	5.814	0.018
s(intake.P,intake.C)	0.000	3.000	0.000	0.426
s(intake.P,intake.F)	0.000	3.000	0.000	0.912
s(intake.C,intake.F)	2.098	3.000	2.523	0.017
PCOS (n = 92)				
s(intake.P)	2.110	2.486	0.642	0.392
s(intake.C)	1.255	1.370	0.523	0.423
s(intake.F)	1.002	1.004	0.552	0.460
s(intake.P,intake.C)	0.000	3.000	0.000	0.382
s(intake.P,intake.F)	0.117	3.000	0.041	0.174
s(intake.C,intake.F)	1.658	3.000	1.273	0.035
**Serum LH (pg/ml) and macronutrient intake**				
Control (n = 88)				
s(intake.P)	1.000	1.000	5.008	0.028
s(intake.C)	1.000	1.000	2.634	0.108
s(intake.F)	1.000	1.000	0.922	0.340
s(intake.P,intake.C)	0.000	3.000	0.000	0.994
s(intake.P,intake.F)	0.000	3.000	0.000	0.568
s(intake.C,intake.F)	0.000	3.000	0.000	0.652
PCOS (n = 92)				
s(intake.P)	1.000	1.000	3.549	0.063
s(intake.C)	1.000	1.000	3.415	0.068
s(intake.F)	1.141	1.267	0.139	0.705
s(intake.P,intake.C)	0.000	3.000	0.000	0.385
s(intake.P,intake.F)	0.001	3.000	0.000	0.372
s(intake.C,intake.F)	0.000	3.000	0.000	0.727
**Number of estrous cycles (n) and macronutrient intake**				
Control (n = 93)				
s(intake.P)	1.015	1.029	1.095	0.310
s(intake.C)	1.000	1.000	1.399	0.237
s(intake.F)	1.000	1.000	0.760	0.383
s(intake.P,intake.C)	0.000	3.000	0.000	0.407
s(intake.P,intake.F)	0.000	3.000	0.000	0.577
s(intake.C,intake.F)	0.000	3.000	0.000	0.630
PCOS (n = 94)				
s(intake.P)	1.000	1.000	8.827	0.003
s(intake.C)	1.000	1.000	11.644	0.001
s(intake.F)	1.000	1.000	0.055	0.815
s(intake.P,intake.C)	1.898	3.000	4.230	0.030
s(intake.P,intake.F)	0.000	3.000	0.000	0.539
s(intake.C,intake.F)	1.448	3.000	1.570	0.236

**Serum triglyceride (mg/dL) and macronutrient intake**				
Control (n = 89)				
s(intake.P)	1.000	1.000	5.591	0.020
s(intake.C)	1.000	1.000	0.965	0.329
s(intake.F)	1.167	1.299	2.463	0.091
s(intake.P,intake.C)	0.000	3.000	0.000	0.624
s(intake.P,intake.F)	0.000	3.000	0.000	0.554
s(intake.C,intake.F)	0.308	3.000	0.116	0.301
PCOS (n = 94)				
s(intake.P)	2.588	2.847	2.143	0.149
s(intake.C)	1.000	1.000	1.214	0.274
s(intake.F)	1.000	1.000	0.567	0.454
s(intake.P,intake.C)	1.286	3.000	1.529	0.028
s(intake.P,intake.F)	0.000	3.000	0.000	0.528
s(intake.C,intake.F)	0.000	3.000	0.000	0.332
**Serum GTT AUC (mmol/L x mins) and macronutrient intake**				
Control (n = 93)				
s(intake.P)	1.000	1.000	1.062	0.306
s(intake.C)	1.154	1.284	0.237	0.775
s(intake.F)	1.001	1.001	8.520	0.004
s(intake.P,intake.C)	0.001	3.000	0.000	0.354
s(intake.P,intake.F)	1.793	3.000	1.638	0.051
s(intake.C,intake.F)	0.002	3.000	0.000	0.316
PCOS (n = 94)				
s(intake.P)	1.796	2.176	1.012	0.382
s(intake.C)	1.000	1.000	0.454	0.502
s(intake.F)	1.000	1.000	0.138	0.711
s(intake.P,intake.C)	0.000	3.000	0.000	0.588
s(intake.P,intake.F)	0.001	3.000	0.000	0.974
s(intake.C,intake.F)	0.000	3.000	0.000	0.798

Supplementary Table 3. Coefficients of the AIC-favored-mixture models associated with energy and food intake. n = sample size (number of mice).

Response Measured	Estimate	Std. Error	t value	df	p-value
**Energy Intake (kJ/day)**					
Control (n = 93)					
diet.P	43.218	9.562	4.520	87.000	0.000
diet.C	85.405	6.059	14.095	87.000	0.000
diet.F	68.224	8.212	8.308	87.000	0.000
diet.P,diet.C	-98.544	26.308	-3.746	87.000	0.000
diet.P,diet.F	-51.366	30.414	-1.689	87.000	0.095
diet.C,diet.F	-132.667	29.338	-4.522	87.000	0.000
PCOS (n = 94)					
diet.P	42.261	6.586	6.417	88.000	0.000
diet.C	59.498	4.174	14.256	88.000	0.000
diet.F	74.233	5.657	13.122	88.000	0.000
diet.P,diet.C	-23.685	18.135	-1.306	88.000	0.195
diet.P,diet.F	-109.096	20.944	-5.209	88.000	0.000
diet.C,diet.F	-76.922	20.198	-3.809	88.000	0.000
**Food Intake (g/day)**					
Control (n = 93)					
diet.P	2.864	0.615	4.656	87.000	0.000
diet.C	5.518	0.390	14.154	87.000	0.000
diet.F	4.299	0.528	8.136	87.000	0.000
diet.P,diet.C	-6.073	1.693	-3.588	87.000	0.001
diet.P,diet.F	-3.368	1.957	-1.721	87.000	0.089
diet.C,diet.F	-8.703	1.888	-4.611	87.000	0.000
PCOS (n = 94)					
diet.P	2.777	0.419	6.620	88.000	0.000
diet.C	3.858	0.266	14.514	88.000	0.000
diet.F	4.661	0.360	12.938	88.000	0.000
diet.P,diet.C	-1.242	1.155	-1.076	88.000	0.285
diet.P,diet.F	-6.958	1.334	-5.217	88.000	0.000
diet.C,diet.F	-5.111	1.286	-3.974	88.000	0.000
