# Supplementary material for: Defining the impact of dietary macronutrient balance on PCOS traits
Source: Nat Commun. 2020 Oct 16;11:5262. doi: 10.1038/s41467-020-19003-5 (PMC7568581; doi:10.1038/s41467-020-19003-5)
Supplement: Supplementary file 3 — Reporting Summary [file 41467_2020_19003_MOESM3_ESM.pdf]

nature research

Corresponding author(s): Associate Professor Kirsty A. Walters

Last updated by author(s): 26/08/2020

Reporting Summary

Nature Research wishes to improve the reproducibility of the work that we publish. This form provides structure for consistency and transparency in reporting. For further information on Nature Research policies, see Authors & Referees and the Editorial Policy Checklist .

Statistics

For all statistical analyses, confirm that the following items are present in the figure legend, table legend, main text, or Methods section.

n/a	Confirmed	
☐	☒	The exact sample size (*n*) for each experimental group/condition, given as a discrete number and unit of measurement
☐	☒	A statement on whether measurements were taken from distinct samples or whether the same sample was measured repeatedly
☐	☒	The statistical test(s) used AND whether they are one- or two-sided<br>*Only common tests should be described solely by name; describe more complex techniques in the Methods section.*
☒	☐	A description of all covariates tested
☐	☒	A description of any assumptions or corrections, such as tests of normality and adjustment for multiple comparisons
☐	☒	A full description of the statistical parameters including central tendency (e.g. means) or other basic estimates (e.g. regression coefficient) AND variation (e.g. standard deviation) or associated estimates of uncertainty (e.g. confidence intervals)
☐	☒	For null hypothesis testing, the test statistic (e.g. *F*, *t*, *r*) with confidence intervals, effect sizes, degrees of freedom and *P* value noted<br>*Give P values as exact values whenever suitable.*
☒	☐	For Bayesian analysis, information on the choice of priors and Markov chain Monte Carlo settings
☒	☐	For hierarchical and complex designs, identification of the appropriate level for tests and full reporting of outcomes
☒	☐	Estimates of effect sizes (e.g. Cohen's *d*, Pearson's *r*), indicating how they were calculated

Our web collection on statistics for biologists contains articles on many of the points above.

Software and code

Policy information about availability of computer code

Data collection	No software was used to collect data.
Data analysis	Data in this paper was an analysed using R (version 3.5.1), Graphpad Prism v8, xPONENT software (Luminex) Multiplex Analyst software Version 5.1 (Luminex) and ImageJ version 1.51 software (NIH). Custom codes used to analyse data in R have been deposited in a publicly accessible database [https://github.com/AlistairMcNairSenior/GFN_PCOS].

For manuscripts utilizing custom algorithms or software that are central to the research but not yet described in published literature, software must be made available to editors/reviewers. We strongly encourage code deposition in a community repository (e.g. GitHub). See the Nature Research guidelines for submitting code & software for further information.

Data

Policy information about availability of data

All manuscripts must include a data availability statement. This statement should provide the following information, where applicable:

- Accession codes, unique identifiers, or web links for publicly available datasets
- A list of figures that have associated raw data
- A description of any restrictions on data availability

Raw data has been deposited in a publicly accessible database (https://github.com/AlistairMcNairSenior/GFN_PCOS).

Field-specific reporting

Please select the one below that is the best fit for your research. If you are not sure, read the appropriate sections before making your selection.

Life sciences study design

All studies must disclose on these points even when the disclosure is negative.

Sample size

Sample size estimates are based on our published data (Caldwell, 2014) for the key PCOS trait of ovulatory dysfunction (reduction in corpora lutea numbers in DHT-induced mouse), assuming two-sided 5% significance and 80% power indicates that 6 mice/group are required. For our analysis reproductive and metabolic responses are mapped onto nutrient intake landscapes allowing quantification of differences in surface topologies using generalised additive modelling (GAM) as previously demonstrated (Solon-Biet, 2015) . The response surface approach using statistical GAM modelling which integrates independent data points, yielding high power for quantifying and comparing surfaces, which reduces the number of mice required. In our previous study we showed significant effects on reproductive function using only 3 mice/group (25 diets) (Solon-Biet, 2015), which gave us 75 (3x25) data points for analysis using GAM. However, as we could not predict how the PCOS mice would respond to the different diets in this study, to further boost power we used 10 mice/diet.

Data exclusions

Twelve animals (6 control and 6 PCOS) that were allocated to diet 3 were euthanized before 10 weeks of experimental diet exposure due to >20% weight loss. Exclusion criteria was pre-established in accordance with animal ethics protocols.

Replication

Mice were set-up in time-staggered cohorts, with each diet treatment spread evenly across cohorts,
to certify there was no biased effect of cohort or batches of mice. Specifically, mice were housed in groups of 2 or 3 mice per cage (4-5 cages per diet) ensuring diet effects were replicated across different cages and cohorts of mice.

Randomization

Female mice were set-up in time-staggered cohorts and were randomly allocated to 2-3 animals per cage. Implant and diet treatments were staggered within and between cohorts to ensure randomization of cohort, implant and diet effects.

Blinding

All samples collected for analysis were assigned a unique ID number, devoid of implant and diet treatment information. All analyses were performed using this number, making sure all sample analyses were blind. For food intake measurements, researchers were required to know treatment allocations as longitudinal food intake measurements required the same diet treatments to be added to each cage throughout the study.

Reporting for specific materials, systems and methods

We require information from authors about some types of materials, experimental systems and methods used in many studies. Here, indicate whether each material, system or method listed is relevant to your study. If you are not sure if a list item applies to your research, read the appropriate section before selecting a response.

Materials & experimental systems

n/a	Involved in the study	
☒	☐	Antibodies
☒	☐	Eukaryotic cell lines
☒	☐	Palaeontology
☐	☒	Animals and other organisms
☒	☐	Human research participants
☒	☐	Clinical data

Methods

n/a	Involved in the study	
☒	☐	ChIP-seq
☒	☐	Flow cytometry
☒	☐	MRI-based neuroimaging

Animals and other organisms

Policy information about studies involving animals; ARRIVE guidelines recommended for reporting animal research

Laboratory animals

This study used 3 week old C57BL/6J female mice (Mus musculus) housed at the ANZAC Research Institute and maintained under standard housing conditions (ad libitum access to food and water in a temperature- and humidity-controlled, 12-h light/dark environment). Specifically, room temperature set point is 22 +/- 1 degrees C and room humidity is between 60-70%. PCOS was induced at three weeks of age and diet treatments started at seven weeks of age.

Wild animals

This study did not utilize wild animals.

Field-collected samples

This study did not collect samples from the field.

Ethics oversight

All experiments were approved by the Sydney Local Health District Animal Welfare Committee within National Health and Medical Research Council guidelines for animal experimentation.

Note that full information on the approval of the study protocol must also be provided in the manuscript.
